# Detection of SARS-CoV-2 antibodies after confirmed Omicron BA.1 and presumed BA.4/5 infections using Abbott ARCHITECT and Panbio assays

**DOI:** 10.1016/j.ijregi.2023.04.014

**Published:** 2023-05-12

**Authors:** Michael Boler, Mark Anderson, Mary Rodgers, Jessica Parumoottil, Ana Olivo, Barbara Harris, Michael Stec, Amy Gosha, Dylan Behun, Vera Holzmayer, Abby Anderson, Ella Greenholt, Tiffany Fortney, Eduardo Almaraz, Gavin Cloherty, Alan Landay, James Moy

**Affiliations:** a Rush University Medical Center, 1725 W Harrison Street Suite 739, Chicago, IL 60612, USA; b Abbott Laboratories, 100 Abbott Park Rd, Abbott Park, IL 60064, USA

**Keywords:** SARS-CoV-2, Antibody, Omicron, Nucleocapsid, Spike, Vaccine

## Abstract

**Background:**

Commercial severe acute respiratory syndrome coronavirus 2 (SARS-CoV-2) antibody tests were developed before variants with spike protein mutations emerged, leading to concerns that these tests have reduced sensitivity for detecting antibody responses in individuals infected with Omicron subvariants. This study was performed to evaluate Abbott ARCHITECT serologic assays, AdviseDx SARS-CoV-2 IgG II, and SARS-CoV-2 IgG for the detection of spike (S) and nucleocapsid (N) IgG antibody increases in vaccinated healthcare workers infected with Omicron subvariants.

**Methods:**

During the BA.1/2 and BA.4/5 waves, 171 SARS-CoV-2-infected individuals (122 in the BA.1/2 wave, 49 in the BA.4/5 wave) were tested for S and N IgG post infection. Sequencing and SARS-CoV-2 variant confirmation were performed on nasal swab samples from individuals infected during the BA.1/2 wave.

**Results:**

Twenty-seven Omicron sequence confirmed individuals in the BA.1/2 wave and all 49 in the BA.4/5 wave had pre-infection antibody data. Compared to pre-infection levels, post-infection S IgG increased 6.6-fold from 1294 ± 302 BAU/ml (mean ± standard error measurement) to 9796 ± 1252 BAU/ml (*P* < 0.001) during the BA.1/2 wave, and 3.6-fold from 1771 ± 351 BAU/ml to 8224 ± 943 BAU/ml (*P* < 0.001) during the BA.4/5 wave. N IgG increased post infection 19.1-fold from 0.2 ± 0.1 to 3.7 ± 0.5 (*P* < 0.001) during the BA.1/2 wave and 13.5-fold from 0.22 ± 0.1 to 3.2 ± 0.3 (*P* < 0.001) during the BA.4/5 wave. Among 159 infection-naïve individuals, positive N IgG levels were detected with a sensitivity of 88% in the 87 individuals who were tested between 14 days and 60 days post infection.

**Conclusions:**

The large increases in post-infection S IgG along with the N IgG sensitivity that was comparable to previously reported N IgG sensitivity data in unvaccinated individuals after Omicron infection, support the use of Abbott SARS-CoV-2 assays for detecting increased S IgG and seroconversion of N IgG in vaccinated individuals post Omicron infection. Given that 68% of the United States population is fully vaccinated, these results are of current relevance.

## Highlights

• S IgG increased 6.6-fold after BA.1/2 infections and 3.6-fold after BA.4/5 infections.
• N IgG increased 19.1-fold after BA.1/2 infections and 13.5-fold after BA.4/5 infections.
• Sensitivity was 77.4% for the detection of post Omicron infection N IgG in vaccinated individuals.
• The detection of S and N IgG increases in vaccinated populations remain sensitive.

## 1 Introduction

Given antigenic differences in the spike protein of the Omicron subvariants compared to the original severe acute respiratory syndrome coronavirus 2 (SARS-CoV-2) sequence [1], there are concerns that commercial spike antibody assays have reduced sensitivity in detecting antibody responses after infection with Omicron subvariants [2,^3^,4]. We previously reported that Abbott antibody assays (ARCHITECT/Alinity SARS-CoV-2 IgG, AdviseDx SARS-CoV-2 IgG II, and Panbio COVID-19 IgG) detected SARS-CoV-2 spike antibodies (S IgG) in 100% (17/17) and nucleocapsid antibodies (N IgG) in 94.1% (16/17) of serum samples from immunocompetent individuals infected with variant B.1.1.7 at 15–26 days after SARS-CoV-2 symptom onset [5]. Since antibody tests were developed before variant of concern lineages with novel mutations in S IgG emerged, it is necessary to confirm that these assays can effectively detect antibody responses after infection with newer SARS-CoV-2 variants of concern.

This study was designed to evaluate the ability of Abbott serologic assays to detect S and N IgG antibody increases in vaccinated healthcare workers infected during two Omicron subvariant waves in the Chicago metropolitan area.

## 2 Study design and methods

This study was approved by the Institutional Review Board of Rush University Medical Center and all individuals provided informed consent to participate in this study. During the Omicron BA.1/2 wave in the Chicago, Illinois metropolitan area (December 2021 to May 2022) [6,7], participants provided a nasal sample for PCR and sequencing within 3 days of a positive SARS-CoV-2 PCR test from December 15, 2021 through April 2022. Individuals returned between 9 days and 150 days (median 58 days) to provide a blood sample for testing S and N IgG. Antibody results of participants in an ongoing longitudinal post-SARS-CoV-2 vaccine-induced antibody study [8] who tested positive for SARS-CoV-2 by PCR or rapid antigen between July and October 2022 (during the BA.4/5 wave) [6] and had their antibody levels tested between 8 days and 97 days (median 53 days) after infection, were used to compare the antibody levels between the two Omicron waves.

For comparison between the previously infected and infection-naïve groups, participants were considered previously infected if they had a history of positive SARS-CoV-2 PCR, positive antigen testing, or positive N IgG results prior to their presumed Omicron infection. Participants were considered infection-naïve if they had a negative history SAR-CoV-2 PCR or antigen testing, and negative N IgG results prior to their presumed Omicron infection.

S and N IgG were measured with the AdviseDx SARS-CoV-2 IgG II (S) and SARS-CoV-2 IgG (N) assays on an Abbott ARCHITECT i2000SR, as described previously. [8] Results are expressed in binding antibody units per milliliter (BAU/ml) for S IgG (values ≥7.1 BAU/ml considered positive) and as the signal/cut-off index (S/C index) for N IgG (values ≥1.4 considered positive). For S IgG, the Abbott AdviseDx SARS-CoV-2 IgG II instrument reported the results in antibody units per milliliter (AU/ml). AU/ml was converted to BAU/ml by multiplying AU/ml by 0.142 (BAU/ml = 0.142 × AU/ml). Genomic sequencing from metagenomic libraries with target enrichment and lineage classification was conducted as described previously [9,10]. For the lateral flow antibody assay, plasma samples were tested using the qualitative Abbott Panbio COVID-19 IgG Rapid Test Device, according to the manufacturer's instructions; this detects S IgG.

The statistical analysis was performed using GraphPad Prism, with the Student paired *t*-test, Chi-square test, and Mann–Whitney *U*-test. Significance was defined as *P* < 0.05.

## 3 Results

All 171 participants had received at least two doses of an mRNA vaccine prior to enrollment into this study. Table 1 shows that the participants were mostly female (85.4%) and White (77.8%); mean age was 40 years.

**Table 1:** Demographic breakdown of the participants by time period of infection.

	Infected during BA.1/2 wave (*n* = 122)	Infected during BA.4/5 wave (*n* = 49)	Overall (*n* = 171)	*P*-value
Age (years), mean ± SD (range)	39.3 ± 10.7 (22–68)	41.8 ± 11.0 (23–66)	40.0 ± 10.8 (22–68)	0.18^a^
Sex, *n* (%)				0.09^b^
Female	108 (88.5%)	38 (77.6%)	146 (85.4%)	
Male	14 (11.5%)	11 (22.4%)	25 (14.6%)	
Race, *n* (%)				
Asian	9 (7.4%)	3 (6.1%)	12 (7.0%)	
Black or African American	11 (9.0%)	2 (4.1%)	13 (7.6%)	
White	89 (72.9%)	44 (89.8%)	133 (77.8%)	
Native Hawaiian or Pacific Islander	1 (0.8%)	0 (0%)	1 (0.6%)	
Other	8 (6.6%)	0 (0%)	8 (4.7%)	
Unknown	4 (3.3%)	0 (0%)	4 (2.3%)	
Vaccine brand, *n* (%)				
Pfizer	120 (98.4%)	49 (100%)	169 (98.8%)	
Moderna	2 (1.6%)	0 (0%)	2 (1.2%)	

SD, standard deviation.

a *t*-test with Welch correction: *P* = 0.18 comparing age between the BA.1/2 and BA.4/5 waves.

b Fisher's exact test: *P* = 0.09 comparing the sex ratio between the BA.1/2 and BA.4/5 waves.

Table 2 shows that during the BA.1/2 wave, 122 nasal samples were collected, of which 72 had a sufficient viral load to be sequenced. Seventy-one were sequenced to be Omicron (63 BA.1, eight unassigned) and one to be Delta. For the 70 confirmed Omicron-infected individuals (one did not return for antibody testing), post-infection S IgG was 11 908 ± 1199 BAU/ml (mean ± standard error of the mean) and N IgG was 3.1 ± 0.3 (index). For the 50 participants for whom a genomic sequence could not be determined, post-infection S IgG (10 985 ± 1402 BAU/ml) and N IgG (3.2 ± 0.3) revealed no differences compared to the 63 BA.1 sequence-confirmed participants (*P* > 0.05, for S IgG and for N IgG). For the one sequenced Delta variant, S IgG was 6522 BAU/ml and N IgG was 5.7.

**Table 2:** Details of the S IgG and N IgG results for participants infected during the BA.1/2 wave.

Wave	Number of participants	S IgG (BAU/ml) Mean ± SEM	N IgG (index) Mean ± SEM
BA.1/2	122		
Blood samples available	121	11 649 ± 903	3.2 ± 0.2
Sequenced confirmed Omicron	71 (70 with blood samples, 1 without blood sample)	11 908 ± 1199	3.1 ± 0.3
Insufficient viral load for sequencing	50	10 985 ± 1402	3.2 ± 0.3
Sequenced confirmed Delta	1	6522	5.7

S, spike; N, nucleocapsid; SEM, standard error of the mean.

**Figure 1:**
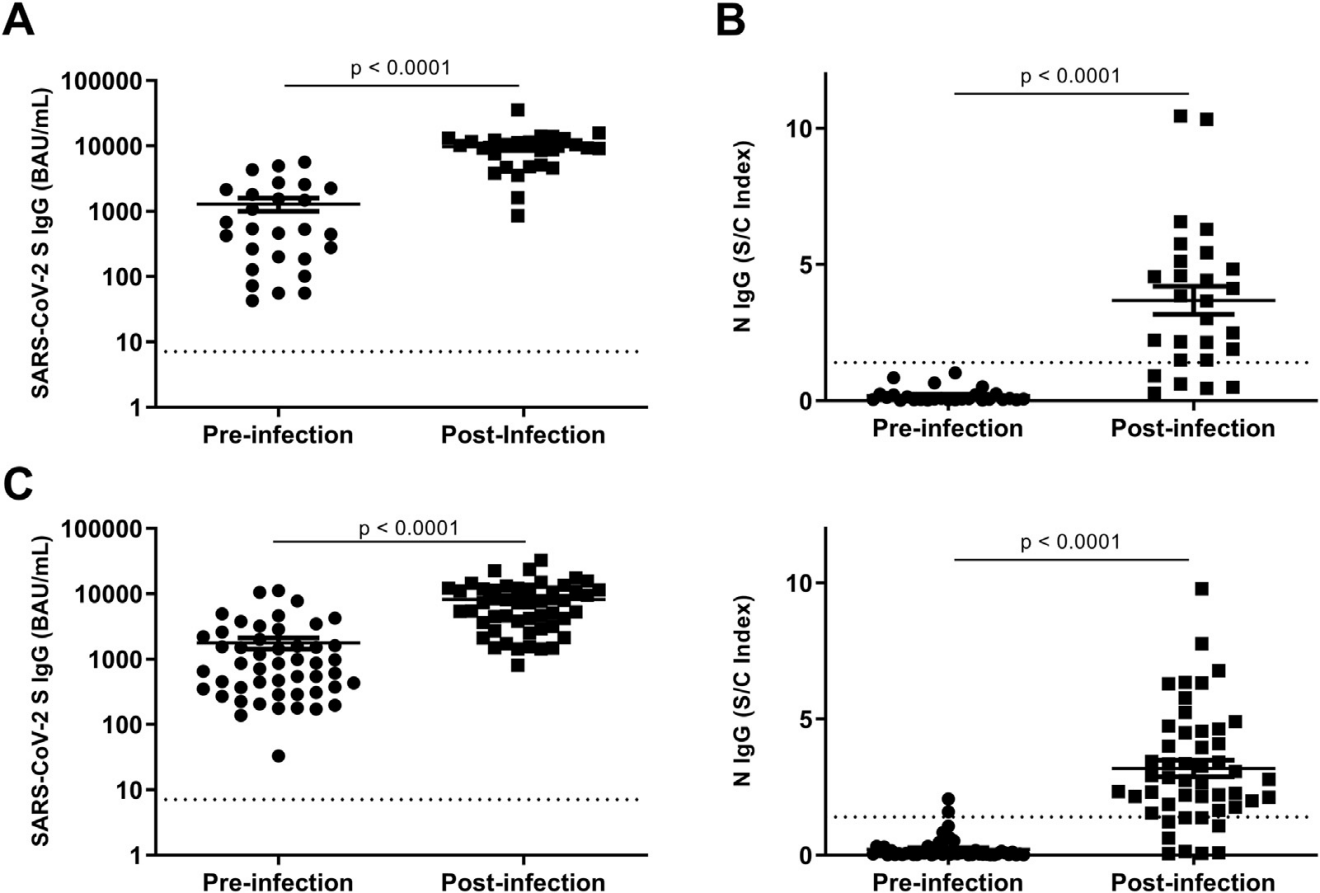
(A) Pre-infection versus post-infection S IgG during the BA.1/2 wave. (B) Pre-infection versus post-infection N IgG during the BA.1/2 wave. (C) Pre-infection versus post-infection S IgG during the BA.4/5 wave. (D) Pre-infection versus post-infection N IgG during the BA.4/5 wave. Horizontal dotted lines indicate S IgG and N IgG assay limits of detection. The statistical analysis was performed using the Mann–Whitney *U*-test, with *P* < 0.05 considered significant.

From their participation in the ongoing post vaccine longitudinal study [8], 27 confirmed Omicron-infected individuals had antibody levels tested prior to their infection (26–183 days from time of infection to time of antibody testing, median 97 days). Post-infection S IgG (9796 ± 1252 BAU/ml) was 6.6-fold higher (*P* < 0.001) than pre-infection levels (1294 ± 302 BAU/ml) (Figure 1A), and post-infection N IgG (3.7 ± 0.5) was 19.1-fold higher (*P* < 0.001) than pre-infection levels (0.2 ± 0.1) (Figure 1B).

During the BA.4/5 wave, 49 individuals were identified in the longitudinal vaccine antibody study who tested positive for SARS-CoV-2 by PCR (*n* = 17) or rapid antigen testing (*n* = 32). All 49 individuals had pre-infection antibody data (6–96 days from time of infection to time of antibody testing, median 34 days). Post infection, S IgG increased 3.6-fold from 1771 ± 351 BAU/ml to 8224 ± 943 BAU/ml (*P* < 0.001) (Figure 1C) and N IgG levels increased 13.5-fold from 0.22 ± 0.1 to 3.2 ± 0.3 (*P* < 0.001) (Figure 1D). Post-infection S IgG levels for the BA.1/2 wave were 1.4-fold higher than the BA.4/5 wave levels (*P* = 0.01), but the post-infection N IgG levels did not differ significantly between the two waves (*P* > 0.05).

Spearman's correlation test was performed to determine whether post-infection S IgG levels correlated with post-infection N IgG levels for both Omicron waves. There was no correlation between S IgG levels and N IgG levels post infection for the BA.1/2 wave or for the BA.4/5 wave (*r* = 0.38, *P* = 0.053 and *r* = 0.08, *P* = 0.57, respectively).

It was next investigated whether participants who had SARS-CoV-2 infections prior to their Omicron infection responded differently in terms of S IgG compared to infection-naïve participants. Of the 99 participants (from both Omicron waves) who had pre- and post-Omicron infection antibody data, 12 (six in each wave) had a SARS-CoV-2 infection prior to enrolling in this study. Ten of 12 (83.3%) previously infected individuals compared to 85 of 87 (97.7%) naïve individuals had increased S IgG post Omicron infection (*P* = 0.02, Chi-square). During the BA.1/2 wave, post-infection S IgG levels did not differ significantly between previously infected (11 715 ± 8075 BAU/ml) and naïve (11 645 ± 877 BAU/ml) participants (*P* = 0.99). The difference in N IgG levels between previously infected (4.9 ± 1.8) and infection-naïve (3.1 ± 0.2) participants was also not significant (*P* = 0.053). During the BA.4/5 wave, post-infection S IgG levels did not differ significantly between previously infected (3816 ± 1471 BAU/ml) and naïve (8840 ± 1024 BAU/ml) participants (*P* = 0.08) . However, N IgG levels were significantly higher in previously infected participants (5.0 ± 1.1) compared to infection-naïve participants (2.9 ± 0.3) (*P* = 0.02) . These statistical comparisons might not be representative of a larger population given the small sample size of the previously infected populations (six for each wave).

The sensitivity of N IgG in detecting Omicron infections was evaluated. Among the 171 individuals in the study, 134 (78.4%) had post-infection positive N IgG. The sensitivity increased to 88.5% (77/87) when tested 14–60 days post infection. Among the 159 infection-naïve individuals, sensitivity was 77.4%. The sensitivity increased to 88% (66/75) when tested 14–60 days post infection. Among the 12 previously infected individuals, four had pre-infection positive N IgG and three of these four (75%) had an increase of ≥1.4 (S/C index) post infection. Eight had pre-infection N IgG <1.4 index and seven of these eight (87.5%) seroconverted. Table 3 shows pre- and post-infection S and N IgG data for the 12 previously infected individuals.

**Table 3:** Antibody details of 12 previously infected individuals^a^.

Omicron wave	Pre-infection S IgG (BAU/ml)	Post-infection S IgG (BAU/ml)	Pre-infection N IgG (index)	Post-infection N IgG (index)	Time from infection to antibody testing (days)
BA.1/2	680	4593	1.02	10.45	19
BA.1/2	73	3830	0.01	2.22	16
BA.1/2	200	1607	0.51	10.33	30
BA.1/2^	48^	35^	0.14^	0.12^	28
BA.1/2#	2692#	51 651#	2.14#	3.00#	50
BA.1/2	2117	8576	1.69	3.28	36
BA.4/5	3783	3803	0.83	3.26	79
BA.4/5*	1999*	1474*	0.11*	6.77*	62
BA.4/5	446	3636	0.32	2.28	9
BA.4/5	979	1716	0.16	9.79	97
BA.4/5	3458	10 838	2.07	4.01	37
BA.4/5	197	1430	1.59	4.09	36

S, spike; N, nucleocapsid; BAU, binding antibody units.

a Of the two individuals who did not have a post-infection increase in S IgG, one (^) also did not have an increase in N IgG, while the other (*) had an increase in N IgG. One individual (#) had an increase in S IgG, but N IgG did not increase by ≥1.4.

A subset of 35 participants during the BA.1/2 wave and 38 participants during the BA.4/5 wave had their post-infection plasma tested with the Panbio lateral flow anti-S antibody assay. All 73 samples were positive on the Panbio assay. The S IgG for these participants ranged from 613 BAU/ml to 54 175 BAU/ml.

## 4 Discussion

We previously reported that vaccinated individuals infected during the Omicron BA.1/2 wave had significantly higher S IgG (8304 BAU/ml) than individuals infected prior to the Omicron BA.1/2 wave (3740 BAU/ml) [8]. The current study showed that sequence-confirmed Omicron BA.1 infections led to high levels of S IgG (11 908 BAU/ml), supporting the previously reported observation of high post-infection S IgG in presumed Omicron-infected individuals. This study demonstrated a 6.6-fold increase in S IgG after infection, which was also observed during the BA.4/5 wave, where mean S IgG levels increased 3.6-fold after infection. Importantly, 97.7% and 83.3% of naïve and previously infected individuals, respectively, had increased post-Omicron infection S IgG levels. These data demonstrate that the Abbott S IgG assay detects large increases in antibodies after Omicron SARS-CoV-2 infections despite the multiple spike mutations [1] in the subvariants.

Post-Omicron infection positive N IgG was detected with a sensitivity of 77.4% (88% when tested 14–60 days post infection) among the infection-naïve vaccinated individuals. These findings are similar to a previously reported sensitivity of 84% in 37 infection-naïve unvaccinated individuals [3]. N IgG increases of ≥1.4 index in 10 out of 12 previously infected individuals were detected. Due to 68% [11] of the population in the United States being vaccinated, it is believed that the study results are more relevant than those reported in previous publications focusing on unvaccinated individuals [2,^3^,12].

Study limitations include a limited study size with a single cohort of participants. This cohort was of low demographic diversity and thus the results cannot be generalized to male individuals and non-White populations. Viral samples during the Omicron BA.4/5 wave were not available for sequencing; however, during this time >75% of Chicago area [6] SARS-CoV-2 cases were Omicron BA.4/5. Another limitation is the low percentage (12%) of previously infected participants; thus, statistical comparisons between previously infected and naïve cohorts could not be conducted. Although all 72 post-infection plasma samples tested positive with the qualitative Panbio rapid test, it cannot be determined whether the test detected new antibodies from the Omicron infections, because all participants were vaccinated and had anti-S antibodies prior to infection.

This work demonstrates that previous observations of reduced antibody detection among vaccine/infection-naïve individuals who were infected with Omicron [2,^3^,12] might not extend to vaccinated individuals with breakthrough infections. For vaccinated individuals, the results demonstrate that the Abbott SARS-CoV-2 S IgG (when pre-infection levels are available) and N IgG assays are useful for the detection of Omicron infections.

## Conflict of interest

The authors declare the following financial interests/personal relationships: MA, MR, AO, BH, MS, VH, AA, EG, TF, EA, and GC are employees and shareholders of Abbott Laboratories. This work was funded by Abbott Laboratories. AL is a consultant for Abbott Laboratories.
